# Osteoid cell-derived chemokines drive bone-metastatic prostate cancer

**DOI:** 10.3389/fonc.2023.1100585

**Published:** 2023-03-21

**Authors:** Catherine S. Johnson, Leah M. Cook

**Affiliations:** ^1^ Department of Pathology and Microbiology, University of Nebraska Medical Center, Omaha, NE, United States; ^2^ Eppley Institute for Research in Cancer and Allied Diseases, Omaha, NE, United States; ^3^ Fred & Pamela Buffett Cancer Center, University of Nebraska Medical Center, Omaha, NE, United States

**Keywords:** bone, prostate, cancer, metastasis, chemokine, therapy

## Abstract

One of the greatest challenges in improving prostate cancer (PCa) survival is in designing new therapies to effectively target bone metastases. PCa regulation of the bone environment has been well characterized; however, bone-targeted therapies have little impact on patient survival, demonstrating a need for understanding the complexities of the tumor-bone environment. Many factors contribute to creating a favorable microenvironment for prostate tumors in bone, including cell signaling proteins produced by osteoid cells. Specifically, there has been extensive evidence from both past and recent studies that emphasize the importance of chemokine signaling in promoting PCa progression in the bone environment. Chemokine-focused strategies present promising therapeutic options for treating bone metastasis. These signaling pathways are complex, with many being produced by (and exerting effects on) a plethora of different cell types, including stromal and tumor cells of the prostate tumor-bone microenvironment. ​This review highlights an underappreciated molecular family that should be interrogated for treatment of bone metastatic prostate cancer (BM-PCa).

## Introduction

PCa is the second most common cancer, is the second leading cause of cancer death in American men, and over 34,000 men are predicted to die of PCa in 2023 (1). Up to 90% of advanced prostate cancers metastasize to bone, and there are currently no curative therapies for BM-PCa (2). Interactions between BM-PCa cells and cells of the bone microenvironment cooperate to drive tumor growth in bone. Specifically, BM-PCa cells are known to produce factors that alter the normal bone remodeling cycle (3, 4).

During normal bone remodeling, mononuclear cells fuse to form multinuclear osteoclasts which secrete hydrogen ions and hydrolytic enzymes, including proteases such as collagenase and cathepsin k, to induce acid and enzyme-mediated bone resorption (5, 6). This releases bone-sequestered growth factors, including transforming growth factor-beta (TGF-β) and insulin-like growth factor (IGF)-1, and stimulates the differentiation of mesenchymal stem cells into bone-forming osteoblasts, which secrete extracellular matrix proteins and deposit hydroxyapatite mineral to generate new bone matrix (5, 6). These processes of osteolysis and osteogenesis are coupled, with bone resorption promoting the differentiation of MSCs into osteoblasts and osteoblast-derived factors, specifically receptor activator of nuclear factor-kappa B ligand (RANKL), driving osteoclastogenesis. This coupling allows these two processes to balance each other and the net bone mass to remain constant (5, 6).

PCa cells in the bone microenvironment disrupt this balance by: 1) over-stimulating osteoblasts to produce new bone, resulting in the production of a weaker woven type of newly formed bone, and 2) by promoting osteoclastogenesis, which leads to excessive osteolysis and the release of large amounts of bone-sequestered growth factors, such as TGF-β, which promote BM-PCa disease progression (3, 4, 7, 8). This hijacking of the normal bone remodeling process not only drives tumor growth in bone, but also leads to skeletal related adverse events such as pain, spinal cord compression and increased risk of fractures from the excessive bone resorption and increased frailty of cancer-induced bone (2, 9, 10).

Approved therapies, such as bisphosphonates and Denosumab (a monoclonal antibody to RANKL), target osteoclasts either by inducing of osteoclast cell death or by inhibiting osteoclastogenesis, respectively (11, 12). While multiple targeted therapies, including osteoclast-targeted therapies have been designed to block this process of tumor-induced bone remodeling, these have failed to extend patient survival (13). Thus, a greater understanding of the crosstalk between BM-PCa cells and other osteoid cells is necessaru to design effective new therapies.

A unique feature of PCa, in contrast to other types of bone metastasis, excessive osteosclerosis regulated by interations with osteoid cells, such as mesenchymal stromal cells, osteoblasts, and osteocytes. Mesenchymal stromal cells (MSCs) were originally isolated from bone marrow by Friedenstein and characterized as an adherent fibroblast-like cell type capable of maturing into bone-forming cells (14). MSCs have been classified as multipotent adult stem cells capable of differentiating along three mesodermal lineages into adipocytes, osteocytes, and chondrocytes and are therefore also known as mesenchymal stem cells (15–17). Studies have observed significant heterogeneity in populations of MSCs, including differences in growth rates, gene expression, and differentiation potential with variability attributed to donor age, tissue source, culture conditions, and other still unidentified genetic and environmental factors (16, 18). In the bone microenvironment, MSCs are important in the process of osteogenesis by differentiating into osteoblasts. Osteoblasts are active bone matrix-secreting cells. Osteoblasts sit on nonmineralized surfaces in bone and secrete extracellular matrix proteins, hydroxyapatite for matrix mineralization, and growth factors and cytokines that stimulate new bone formation. Eventually, osteoblasts become either bone lining cells or mature osteocytes embedded in the bone matrix they have produced, where they coordinate responses to mechanical stress by stimulating bone resorption and replacement (19).

BM-PCa cells are known to interact with all three of these types of osteoid cells, and many different factors derived from these bone cells have been characterized to drive BM-PCa progression (20–26). While these include a variety of growth factors and cytokines, this review will specifically highlight the impact of a subtype of cytokines known as chemokines, found in abundance in the tumor microenvironment.

Studies show that MSCs, osteoblasts, and osteocytes produce many different types of chemokines, and their expression of several chemokines is altered following exposure to BM-PCa cells (20, 27–29). Despite substantial evidence of the importance of chemokines, the complexity of their functions has made clinical translation difficult. Nonetheless, this review aims to re-introduce the possibility of targeting these chemokines and their receptors in the treatment of BM-PCa patients.

## Osteoid-derived chemokines in BM-PCa

**Figure 1:**
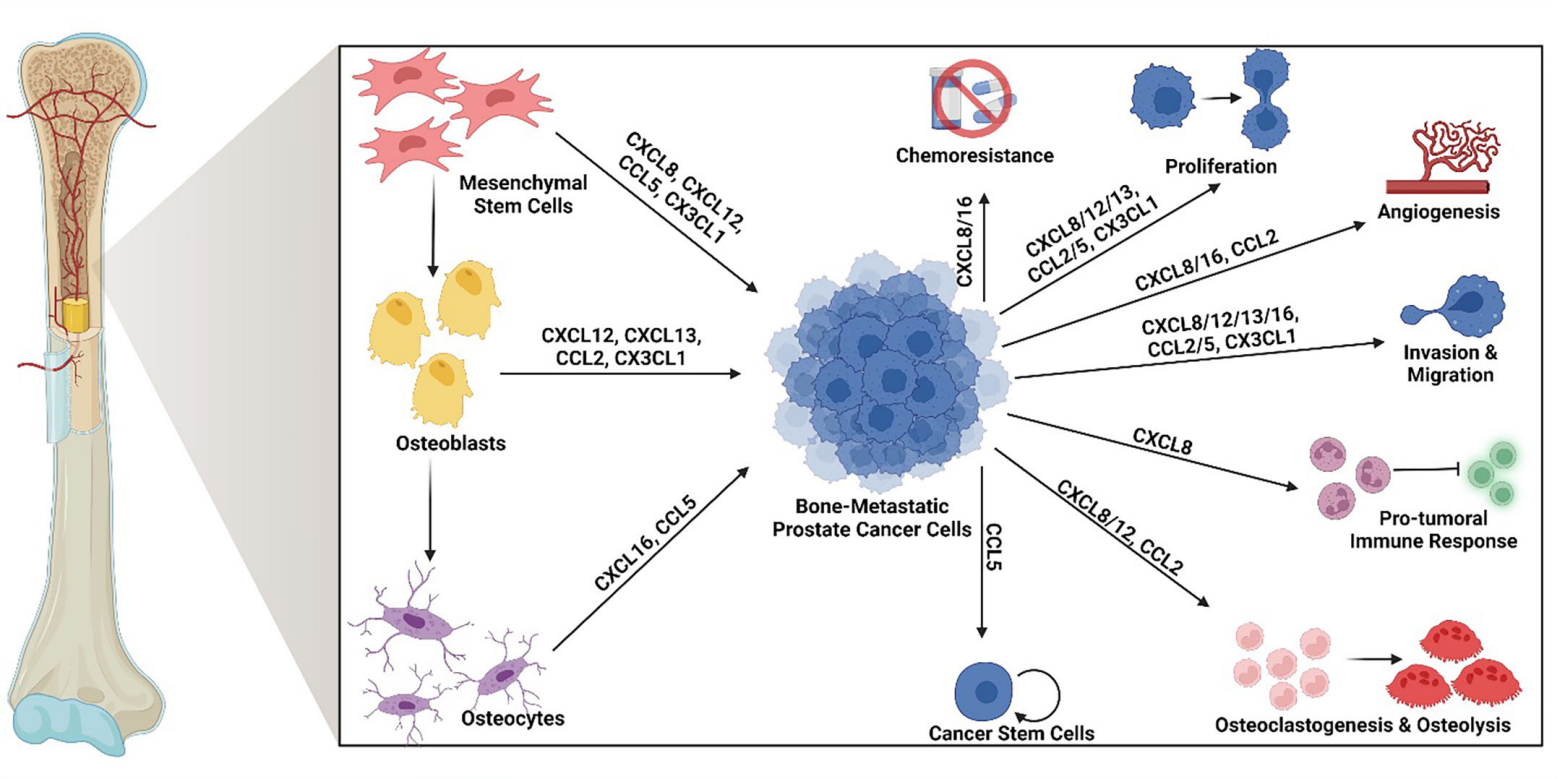
Osteoid cell-derived chemokines and their roles in bone-metastatic prostate cancer.​ Many chemokines produced by osteoid cells in the bone promote bone-metastatic prostate cancer progression. Specifically, mesenchymal stem cells produce CXCL8, CXCL12, CCL5, and CX3CL1; osteoblasts produce CXCL12, CXCL13, CCL2, and CX3CL1, and osteocytes produce CXCL16 and CCL5. Among these, CXCL8 and CXCL16 have been found to promote chemoresistance. CXCL8, CXCL12, CXCL13, CCL2, CCL5 and CX3CL1 enhance tumor cell proliferation. CXCL8, CXCL16, and CCL2 contribute tumor-induced angiogenesis. CXCL8, CXCL12, CXCL13, CXCL16, CCL2, CCL5, and CX3CL1 induce tumor cell migration and invasion. CXCL8 drives a pro-tumoral immune response, often involving myeloid cell suppression of anti-tumor effector cells. CXCL8, CXCL12, and CCL2 induce osteoclastogenesis and tumor-induced osteolysis. CCL5 increases the population of cancer stem cells. Created by Biorender.com.

Chemokines are small cytokine proteins classically characterized by their ability to induce leukocyte migration *via* G protein coupled receptor signaling. This large family of signaling ligands currently consists of 50 known ligands and is divided into four families (XC, CXC, CC, CX3C) based on the position of conserved cysteine residues (27, 30, 31). Although initially classified as chemokines based on their chemotactic functions, many of these signaling ligands have roles in several cellular processes, including bone remodeling, angiogenesis, as well as tumor cell proliferation, survival, invasion, migration, stemness, chemoresistance, and immune evasion (32–37). The chemokines discussed in below have been shown to play important roles in driving BM-PCa growth, survival, migration & invasion, chemoresistance, and tumor-induced osteolysis (summarized in **Figure 1**).

CXCL8: CXCL8 (also known as interleukin-8), is a pro-inflammatory chemokine originally identified as a neutrophil chemotactic factor that signals through CXCR1 and CXCR2 to induce neutrophil migration out of the bone marrow and toward sites of injury and inflammation (38, 39). In the context of BM-PCa, CXCL8 was shown to be upregulated in MSCs in response to BM-PCa cells (28). Likewise, CXCL8 also directly promotes osteoclastogenesis (40, 41) in support of extensive evidence that it is important in PCa-induced bone remodeling. Further, in PCa patients, high serum levels of CXCL8 positively correlated with higher Gleason score and disease progression (42, 43). Additionally, one study found high serum CXCL8 correlated with the presence of bone metastases in patients (44). Higher PCa patient serum CXCL8 levels were also associated with an increase in T-cell suppressive myeloid-derived suppressor cells (MDSCs) (45).

Preclinical studies in BM-PCa revealed that CXCL8 promotes *in vivo* tumor growth and angiogenesis of PC3, an osteolytic PCa cell line derived from a human bone metastasis (46, 47). *In vitro* studies showed CXCL8 to promote BM-PCa cell migration and PCa-induced osteoclastogenesis (48–50). Another group looking at factors overexpressed by an osteogenic PCa xenograft identified CXCL8, which suggests a role for CXCL8 in osteogenesis in addition to its previously found role in promoting osteolysis (51). In another study, CXCL8 upregulated PCa cell expression of bone sialoprotein (BSP), an extracellular bone matrix protein, and increased PCa invasion and adhesion to bone chips *in vitro*, showing PCa cells can contribute to bone remodeling by producing bone matrix proteins and that CXCL8 has a role in this process as well as in the process of PCa metastasis to bone (52). The role of CXCL8 in bone remodeling and tumor progression makes it ideal for therapeutic targeting.

CXCL12: CXCL12 (or Stromal Derived Factor-1α (SDF-1α)) is best known for signaling the retention of hematopoietic stem cells and innate immune cells in the bone marrow niche through its receptor CXCR4 (53, 54). Multiple studies have also shown recruitment of osteoclast pre-cursors by CXCL12, which upregulated the secretion of osteolytic enzymes and led to increased bone resorption (55–57). In addition to its role in osteolysis, CXCL12/CXCR4 signaling is important in the differentiation of MSCs into osteoblasts, with targeted genetic deletion of either the receptor CXCR4 or its ligand CXCL12 resulting in impaired osteoblast differentiation and decreased bone formation in mouse models (58–61).

CXCL12 may also have an indirect impact on PCa migration, invasion, and angiogenesis through altered gene expression of matrix metalloproteinases, including of MMP-1, MMP-3, MMP-9, and MMP-14 in PCa cells (62, 63). Overexpression of MMP-1/collagenase-1 in PCa cells has been shown to increase migration *in vitro* and incidence of metastasis in mouse models (64–66). Genetic silencing of MMP-3, also referred to as stromelysin-1, reduced tumor progression and angiogenesis in mouse models of BM-PCa, and MMP-3 has been shown to play a role in migration in other cancer types, including breast cancer and osteosarcoma (67–69). Inhibition of MMP-9/gelatinase B reduced PCa invasion *in vitro* and tumor growth, invasion, and angiogenesis *in vivo* (70–72). MMP-14 is reported to be important in the migration of multiple types of cancer, with mutations in MMP-14 leading to a decrease in PCa migration *in vitro* and peptide inhibitors of MMP-14 decreasing breast cancer invasion *in vitro* and metastasis *in vivo* (73, 74). These studies suggest CXCL12 promotes PCa migration, invasion, and angiogenesis *via* altered MMP gene expression, in addition to the direct chemotactic effect of CXCL12 on PCa cells.

CXCL12 also plays an important role in PCa in the bone with increased expression of CXCR4 observed in bone-metastatic PCa cell lines and patient samples. Further, in patients with metastatic PCa, high CXCR4 expression correlated with decreased survival (75). Pertinent to BM-PCa, CXCR4 is upregulated in PCa cells cultured with bone stromal cells (76). Additionally, CXCL12 is expressed by osteoblasts and may mediate the increased homing of PCa cells to osteoblast-rich regions of the bone, such as the lateral endocortical surfaces (77, 78). Inhibition of CXCR4 blocked the increase in number of BM-PCa cells found in osteoblast-dense niches in mouse tibia following intracardiac injection, suggesting that CXCL12/CXCR4 signaling may play an important role in PCa metastasis to bone (78). Likewise, *in vivo* treatment with CXCR4 antagonists reduced intratibial growth and tumor-induced osteolysis of PC3 cells, whereas overexpression of CXCR4 increased PC3 tumor growth in bone and osteolysis (76, 79, 80).

MSCs also produce CXCL12, which can induce PCa cell migration *in vitro* (62, 63, 77, 81, 82). A previous study suggested a role for CXCL12 in the creation of a pre-metastatic niche in bone through the upregulation of CXCL12 expression in MSCs by PCa exosome-derived pyruvate kinase M2 (PKM2) and demonstrated a CXCR4-dependent increase in metastatic seeding and growth of PC3 cells in mice treated with PCa exosomes (83). Collectively, these findings demonstrate the CXCL12/CXCR4 signaling axis to be a viable target for treating PCa growth in bone.

CCL2: One of the most studied chemokines, CCL2 (or Monocyte Chemoattractant Protein 1 (MCP-1)), was named for its ability to recruit monocytes to sites of injury or infection; however, dendritic cells, memory T cells, basophils, and NK cells can be recruited by this pro-inflammatory chemokine through signaling *via* its receptor CCR2 (84–90). Several different cell types produce CCL2, including monocytes, endothelial cells, epithelial cells, smooth muscle cells, fibroblasts, and osteoblasts (91–95).

In addition to its role in inflammation, CCL2 also has an important role in bone remodeling. Given that monocytes are precursors for osteoclasts and that CCL2 is important for monocyte recruitment, it is not surprising that CCL2 and CCR2 KO mice have decreased numbers of osteoclasts, which resulted in decreased bone resorption and increased bone mass (96–99). However, other studies suggest the role of CCL2 in bone remodeling may be more complex. One study found that mechanical stress upregulated CCL2 expression in osteoblasts *via* ERK1/2 signaling and led to increased bone formation in mice, suggesting CCL2 may also be involved in osteogenesis in addition to osteoclastogenesis (95). Osteoblast expression of CCL2 was discovered to be induced by a number of different factors including growth differentiation factor-15 (GDF15), a member of the TGF-β family of ligands that is highly expressed by PCa cells. High serum GDF15 levels are associated with bone metastasis, suggesting that GDF15 may contribute to high levels of CCL2 production by osteoblasts in BM-PCa (29, 100). Another factor produced by PCa cells that can upregulate CCL2 is parathyroid hormone-related protein (PTHrP), which increases CCL2 expression in osteoblasts *via* the transcription factors C/EBPβ and NFκB (101). These studies suggest that PCa cells in the bone microenvironment manipulate osteoblast expression of CCL2 to drive tumor-induced bone remodeling.

Multiple studies show CCL2 recruits monocytes to BM-PCa tumors and increases the number of tumor-associated osteoclasts, such that CCL2 inhibition decreases tumor-induced bone resorption in mice (102, 103). Recruited monocytes were also found to mature into tumor-associated macrophages (TAMs), which promote cancer progression through multiple mechanisms (102, 104–106), including promotion of tumor proliferation, cancer cell stemness, metastasis, and therapy resistance, while also suppressing anti-tumor immune responses (107–111).

In patients, CCR2 expression levels positively correlate with increasing PCa stage, and CCL2 expression is associated with reduced survival (101, 112). Additionally, serum CCL2 levels were increased in those with bone metastases compared to localized PCa (101). These findings demonstrate that CCL2 may play a particularly key role in PCa bone metastases, where it can contribute to tumor growth, invasion, angiogenesis, bone remodeling, and recruitment of pro-tumoral immune cells.

CCL5: The pro-inflammatory chemokine CCL5, initially named Regulated on Activation, Normal T cell Expressed and Secreted (RANTES), was first identified as a T cell chemoattractant (113). It was later found to recruit monocytes, dendritic cells, NK cells, basophils, eosinophils, and mast cells as well (114–119). CCL5 can bind and signal through many receptors, including CCR1, CCR3, CCR4, and CCR5 (with highest affinity) (120, 121). In addition to its role in inflammation, CCL5 also has a role in bone remodeling and is known to recruit osteoblasts (122). Specifically, total KO of CCL5 in mice results in decreased bone mass, although this phenotype disappears as these mice age, likely through compensatory signaling through other chemokines not identified in this study (99).

Surprisingly, there is little to no evidence of CCL5 in osteoblast function. However, in osteocytes, mechanical pressure was found to upregulate expression of CCL5, which was partially responsible for an increase in BM-PCa *in vitro* growth and invasion by osteocyte-derived factors (123).

In PCa, multiple cell types have been found to increase tumor cell proliferation, migration, invasion, chemoresistance, and cancer stem cell populations *via* secretion of CCL5 (124). *In vivo*, CCR5 inhibitor treatment reduced bone metastasis following intracardiac injection of PCa cells in mice, suggesting CCR5 may be a potential therapeutic target to reduce bone metastasis in PCa patients (125). Further support for the clinical relevance of CCL5 in PCa patients comes from research showing that CCL5 expression is increased in tissue samples from PCa patients compared to healthy controls and that increasing serum CCL5 levels correlate with increasing Gleason score in PCa patients (110). A separate study comparing gene expression from PCa tissue to BPH tissue found increased expression of both CCL5 and CCR5 in PCa samples (126). These findings demonstrate the impact of CCL5 on many processes essential for PCa progression.

CXCL13: CXCL13, also called B Cell Attracting Chemokine 1 (BCA-1), was discovered as a B-cell chemotactic factor important for the homing of B cells to lymphoid organs (127). While CXCL13 was originally found to be derived from dendritic cells and T follicular helper cells, it was later also found to be produced by osteoblasts and human bone marrow endothelial (HBME) cells and to promote MSC osteoblastic differentiation (127–129).

Since its identification, CXCL13 has been revealed to induce PCa proliferation, migration, and invasion (128, 130, 131). Mechanistically, *in vitro* assays showed that CXCL13 promotes PCa proliferation through c-Jun N-terminal kinase (JNK) signaling and invasion *via* protein kinase B (Akt) and extracellular signal-regulated kinase (ERK)-1/2 signaling (130). CXCL13 also promoted BM-PCa adhesion to HBME cells *in vitro*, suggesting a role for CXCL13 in the extravasation of PCa cells during metastasis (127, 128).

Serum CXCL13 levels are increased in patients with PCa compared to healthy controls and patients with benign prostatic hyperplasia (BPH) or high-grade prostatic intraepithelial neoplasia (HGPIN). Notably, CXCL13 is the only ligand for the receptor CXCR5, and CXCR5 expression has been shown to positively correlate with PCa grade (128). CXCL13 expression has also been reported to be upregulated by interleukin 6 (IL-6) through an unknown mechanism, which may be significant in BM-PCa, as IL-6 levels are observed to be higher in PCa patients with bone metastases (128, 132, 133). These studies provide support for an important role for CXCL13 in PCa bone metastases.

CXCL16: CXCL16 is a pro-inflammatory chemokine that can be upregulated by the cytokines interferon-gamma (IFN-γ) and tumor necrosis factor-alpha (TNF-α) and has been shown to recruit CXCR6 expressing T helper 1 (Th1) cells, cytotoxic CD8+ T effector cells, plasma cells, NK cells, and NKT cells (134–140). CXCL16 is produced as a transmembrane protein that is cleaved by a disintegrin and metalloproteinase domain-containing protein 10 (ADAM10) to release the soluble chemokine (135, 141). Its receptor CXCR6, whose only ligand is CXCL16, was previously found to be a co-receptor for HIV infection (142). In some cancers, such as colorectal and renal cancer, CXCL16 is associated with tumor-infiltrating lymphocytes and good prognosis (143–145). However, in PCa, CXCL16 has been linked to cancer progression and metastasis (146). In patients, increased expression of the CXCL16 and CXCR6 were observed in tissue samples of tumors with higher Gleason scores and in metastatic PCa cell lines (147–149).

Several bone stromal cells, including MSCs, monocytes, fibroblasts, and osteocytes have all been shown to express CXCL16 (135, 142, 150–153). In primary tumors, PCa-derived CXCL16 recruits CXCR6-expressing MSCs which upregulate PCa CXCR4 expression, induce EMT markers *via* CXCL12 secretion, and promote tumor growth *in vivo* in a subcutaneous model (154). Intracardiac injection of mouse PCa cells into CXCR6 knockout (KO) mice resulted in reduced bone metastasis compared to wildtype mice, suggesting that CXCR6 has a role in the homing of PCa to bone (154). CXCL16 was also found to induce the expression of pro-angiogenic factors such as vascular endothelial growth factor (VEGF) in BM-PCa cells and to promote chemoresistance to docetaxel (147, 155). BM-PCa cell invasion and VEGF secretion was found to be dependent on AKT/Mammalian Target of Rapamycin (mTOR) signaling (154). These studies bring to light the importance of CXCL6/CXCR6 signaling in driving PCa progression by promoting metastasis, angiogenesis, and chemoresistance.

CX3CL1: CX3CL1 (fractalkine), the only identified member of the CX3C family of chemokines, is produced as a membrane bound protein which, and like CXCL16, can undergo proteolytic cleavage to release a soluble chemotactic domain. It is upregulated by IFN-γ and TNF-α and functions as a chemoattractant for Th1 cells, NK cells, and monocytes that express the receptor CX3CR1 (156–159). In the bone, membrane-bound CX3CL1 is detected on BM-MSCs, osteoblasts, and bone marrow endothelial cells, and the soluble form of cleaved CX3CL1 is detected in bone marrow (160).

CX3CL1 has been shown to have an important role in bone resorption as a chemokine that recruits monocyte osteoclast precursors and promotes osteoclastogenesis (157, 161–165). Treatment of mice with CX3CR1 neutralizing antibody decreased bone resorption *in vivo*, and CX3CR1-deficient pre-osteoclasts failed to differentiate *in vitro*. Osteoblasts also express the receptor CX3CR1, and CX3CL1 has been shown to have an impact on osteoblast differentiation. CX3CR1-deficient osteoblasts have delayed induction of a key osteoblastic transcription factor runt-related transcription factor 2 (RUNX2), but upregulation of a late osteoblastic transcription factor osterix (OSX), as well as decreased calcium deposition *in vitro*, suggesting CX3CL1/CX3CR1 signaling regulates the temporal expression of transcription factors required for normal osteoblast differentiation. CX3CR1-deficient osteoblasts also have reduced expression of osteoclastogenic factor RANKL. Treatment of wildtype osteoblasts with recombinant CX3CL1 induces both RUNX2 and OSX but decreases expression of several bone matrix proteins, including osteonectin, osteocalcin, and osteopontin (161). Thus, the impact of CX3CL1/CX3CR1 signaling in osteoblasts is complex, but it clear that this chemokine plays an important role in bone remodeling.

CX3CR1 is also expressed in prostate epithelial cells. CX3CR1 expression is detected in both normal prostate epithelium and PCa tumors, with higher expression observed in PCa cells compared to healthy prostate epithelial cells (160, 166). Levels of soluble CX3CL1 in the bone marrow were found to be androgen regulated, with the potent androgen dihydrotestosterone upregulating cleavage of CX3CL1 from the plasma membrane of bone cells (160). This may be through upregulation of a disintegrin and metalloproteases (ADAM) enzymes, which have been shown to cleave CX3CL1 and to be upregulated by dihydrotestosterone (167–170). CX3CL1 is suggested to play a role in the extravasation of circulating PCa cells during metastasis to bone. It is expressed on the luminal side of bone marrow endothelial cells, and PC3 cells have been found to adhere to HBME cells *in vitro* under flow conditions recapitulating the shear force measured in microvessels *in vivo*, with this adhesion blocked by CX3CL1 neutralizing antibody (171). CX3CL1 also induces BM-PCa expression of EMT markers and migration and invasion *in vitro* (166, 171, 172). Overexpression of CX3CR1 in BM-PCa cells increased proliferation and decreased apoptosis (166). In mice, intracardiac injection of BM-PCa cells overexpressing CX3CR1 led to increased incidence of spinal metastases compared to mice injected with control BM-PCa cells (166). In PCa patients, serum CX3CL1 levels show a positive correlation with the presence of spinal metastases, suggesting a role for CX3CL1 in the process of PCa metastasis to bone (166, 173).

## Chemokine regulation of bone cancers and bone metastasis

Although we are aware that PCa can progress to development of visceral and soft tissue metastases, bone is the most frequent site of PCa metastasis. Chemokines have are involved in other bone and bone-metastatic cancers as well, suggesting that they may be key for understanding how to target cancer growth specifically in bone. For example, CXCL8 is involved in tumor-induced osteolysis in bone-metastatic breast cancer, and breast cancer cells upregulated osteoblast expression of CXCL8 (41, 174–177). CXCL8 was also increased in serum samples from lung cancer patients with bone metastases and was involved in lung cancer-induced osteoclastogenesis *in vitro* (178, 179). In osteosarcoma, CXCL8 promotes migration and activates Akt signaling to promote survival *in vitro*, and CXCL8 inhibition or knock down (KD) of the receptor CXCR1 decreases lung metastases *in vivo* (180–182). CXCL8 serum levels in patients with multiple myeloma, a cancer originating in bone marrow plasma cells, were elevated compared to healthy controls, and multiple myeloma cells upregulated CXCL8 expression in bone stromal cells (183–186). *In vitro*, CXCL8 promoted multiple myeloma cell survival and tumor-induced osteoclastogenesis (185).

CXCL12 has been reported to recruit multiple types of cancer to bone (187–189). In breast cancer cells, angiopoietin-like protein 2 (ANGPTL2) was found to upregulate CXCR4 and promote bone metastasis in an intracardiac bone metastasis mouse model (190). Among breast cancer cell lines, CXCR4 was more highly expressed by bone metastatic cells (191). In multiple myeloma, CXCL12/CXCR4 signaling has been shown to promote proliferation, migration, invasion, metastasis, chemoresistance, and tumor-induced osteoclastogenesis (192–196). In mice, treatment with CXCR4 antagonist mobilized multiple myeloma cells from the bone marrow and induced cancer cell death (197). In osteosarcoma, CXCL12/CXCR4 signaling promotes cancer progression and metastasis, and CXCR4 expression is associated with poor prognosis in patients (198–204).

CXCL13 and CXCR5 are also overexpressed in breast cancer and associated with increasing stage and metastasis (205, 206). CXCL13/CXCR5 signaling promotes osteosarcoma migration and invasion *in vitro* (207). CXCR5 is also expressed on bone marrow-metastatic neuroblastoma cells, which migrate toward CXCL13 *in vitro*, suggesting CXCL13/CXCR5 signaling may be involved in neuroblastoma metastasis to bone marrow (208).

As seen in PCa, primary breast cancer tumors recruit CXCR6 expressing MSCs *via* CXCL16, and the tumor-associated MSCs then secrete other chemokines, such as CCL5 and CXCL10, that promote invasion and metastasis (209). Lung cancer cells also highly express CXCL16/CXCR6, which promote lung cancer cell migration and invasion *in vitro* and bone metastasis *in vivo* (210–212). In osteosarcoma, CXCL16 was found to be upregulated and to induce EMT markers and promote growth and invasion *in vitro* (213, 214).

CCL2 is associated with increased tumor grade and decreased relapse-free survival in breast cancer patients and promotes bone metastasis and osteolysis in mice (215, 216). Breast cancer cells were also found to upregulate osteoblast expression of CCL2, suggesting that breast cancer cells manipulate osteoid cells in the bone microenvironment to promote osteolysis and tumor progression (177, 217). Lung cancer patients with bone metastases have increased serum CCL2, and shRNA KD of CCL2 in non-small cell lung cancer cells reduced intratibial tumor growth and bone resorption in SCID mice (179). CCL2 also promotes osteosarcoma progression, metastasis, and bone resorption (218–221). In multiple myeloma, CCL2 recruits and polarized macrophages to promote chemoresistance through upregulation of MCP-1-induced protein (MCPIP1) (222, 223).

Last, CX3CL1/CX3CR1 play a role in bone metastasis of other cancers, including breast cancer. Specifically, CX3CR1 can be detected on both healthy and malignant breast epithelial cells, with a greater percentage of cells expressing high levels of CX3CR1 in breast cancer tissues and even higher expression in bone metastases (224–226). Breast cancer cells intracardially injected in total CX3CR1 KO mice had reduced bone metastases compared to wildtype mice, and bone metastases in wildtype mice were reduced with CX3CR1 antagonist treatment (224, 225). Spinal metastases in hepatocellular carcinoma patients also have high CX3CL1 and CX3CR1 expression, and CX3CL1 promoted hepatocellular carcinoma cell migration *in vitro* and spinal metastasis *in vivo* (227). Additionally, CX3CL1 is associated with spinal metastasis in lung cancer patients and correlates with poor overall survival (228, 229) and CX3CL1 is also involved in metastasis of cancers originating in the bone (230). One study observed that osteosarcoma cells have increased expression of CX3CL1 compared to healthy osteoblasts (231). These studies confirm the importance of these chemokines in the tumor bone microenvironment, not just in BM-PCa, but also in other bone and bone-metastatic cancers.

## Chemokine-focused therapy for treating BM-PCa

Chemokines also represent promising new options as PCa biomarkers. Serum CCL2 and CXCL12 were significantly elevated in patients with localized PCa compared to healthy donors, which suggests they may be valuable for diagnosing PCa but not specifically for identifying high grade or metastatic PCa (232, 233). CCL5 has been found to be elevated in the blood of PCa patients and, unlike CCL2, it is associated with higher Gleason grade and metastases (110). CXCL8 was elevated in PCa patients with bone metastases and associated with higher grade tumors and AR loss (44, 234). Serum CXCL13 levels have been found to be elevated in PCa patients and discovered to be a better predictor of PCa than PSA levels (128, 131).

Since the prostate tumor-bone microenvironment is rich in chemokines, blocking pro-tumorigenic signals from chemokines may be an effective treatment for patients with bone metastases. Currently, the only chemokine receptor inhibitors approved for use in patients are a CCR5 antagonist (Maraviroc) to treat HIV, a CCR4 antagonist (Mogamulizumab) to treat mycosis fungoides, and a small molecule CXCR4 antagonist (Plerixafor) (235–237). Plerixafor is currently approved for use in patients with Non-Hodgkin Lymphoma and Multiple Myeloma to mobilize hematopoietic stem cells in preparation for autologous transplant (237). However, both Maraviroc and Plerixafor are currently being tested in clinical trials for multiple types of solid tumors.

A phase 1 clinical trial (NCT01736813) of Maraviroc in colorectal cancer showed it was well tolerated and induced partial responses, including decreased proliferation markers in biopsies and partial remission of lung metastases in previously refractory tumors (238). Another phase 1 clinical trial (NCT03274804) of Maraviroc combined with the immunotherapy Pembrolizumab also reported a safe toxicity profile and prolonged disease stabilization in metastatic colorectal cancer patients (239, 240). This suggests CCR5 antagonist treatment may be a promising approach to reduce tumor growth in other solid tumors in which CCL5 plays a pro-tumoral role, such as PCa, where Maraviroc was shown to reduce bone metastasis of intracardially injected PCa cells in mice (125). Preclinical studies also show promise for CCL5 antagonists in pancreatic cancer and bone-metastatic breast cancer, and a current phase 1 clinical trial (NCT04721301) is investigating the combination of Maraviroc with other immunotherapies in metastatic colorectal and pancreatic cancer patients (241, 242).

Inhibition of CXCR4 also showed promise, with pre-clinical testing of Plerixafor reducing the establishment of bone metastases in bone-metastatic prostate cancer mouse models, yet it failed to impact the growth of pre-established bone metastases (243). This suggests CXCR4 may be particularly important in the process of metastasis to bone, and inhibition may be effective in preventing bone metastasis but ineffective in patients who already have bone metastases. Another pre-clinical study showed Plerixafor sensitized BM-PCa cells to docetaxel, suggesting possible benefits of including CXCR4 inhibitors in combination treatment approaches (244).

Pre-clinical testing of CCL2 inhibition also decreased bone metastatic prostate tumor growth, both alone and in combination with docetaxel (245, 246). Unfortunately, when tested in a clinical trial (NCT00992186), the monoclonal antibody used to target CCL2 failed to induce long-term suppression of serum CCL2, resulting in no effect on tumor growth (247, 248). Therefore, more work needs to be done to develop and test inhibitors of chemokine signaling before these treatments can be moved into the clinic.

*Androgen-targeted therapies and chemokines.* The current standard-of-care therapies for BM-PCa are androgen-targeted interventions. Several chemokines may be uniquely important in PCa due to roles of androgen signaling in the mechanism of their effect on cancer cells. For example, the pro-migratory effect of CCL5 on PCa cells occurs *via* suppression of androgen receptor (AR) by inhibition of AR nuclear translocation and is thus an AR-dependent mechanism (249). Some effects of CXCL12 signaling are also AR-dependent. CXCL12 induces PCa cell proliferation by increasing nuclear accumulation of both AR and its co-regulator steroid receptor coactivator 1 (SRC-1) and upregulating AR/SCR-1-responsive genes (250).

There is also evidence of chemokines acting through suppression of androgen signaling. One study reported that coculture of BM-MSCs with PCa cells upregulated their expression of CCL5, which was shown to block AR nuclear translocation and a resultant increase in cancer stem cells and PCa cell invasiveness (251, 252). In a separate study, suppression of AR signaling was determined to mediate CCL5-induced PCa cell migration towards bone stromal cells derived from a PCa patient bone metastasis (249). Likewise, endothelial cell derived CCL5 was shown to downregulate AR and promote PCa cell invasion *in vitro* and metastasis *in vivo* (253). These findings suggest that several pro-tumoral mechanisms of osteoid cell-derived chemokines are enhanced by anti-androgen therapies and promote therapy resistance.

Androgen signaling is also involved in the regulation of chemokine expression. One study found that AR signaling suppressed CXCL8 expression, with androgen deprivation therapy leading to upregulation of CXCL8 (254). Another study reported that AR signaling upregulates expression of CXCL13, with an androgen responsive element (ARE) identified in its enhancer region (131). Androgen signaling also upregulates cleavage of membrane-bound CX3CL1 to increase levels of the soluble chemokine (160).

Additionally, studies have demonstrated a role for AR in regulating CCL2 expression, as siRNA targeting of AR in PCa cells was observed to upregulate CCL2 *via* STAT3 activation by downregulating an AR-inducible protein inhibitor of activated STAT 3 (PIAS3). Another study found AR inhibitors enzalutamide and bicalutamide upregulated CCL2 *via* STAT3 in PCa cells, leading them to propose the use of CCL2/CCR2 or STAT3 inhibitors in combination with AR inhibitors to block the pro-tumoral effects of ADT-induced CCL2 (255). Therefore, targeting of these androgen-regulated chemokines may be especially effective in PCa patients.

*Bone-focused therapies and chemokine regulation.* In addition to androgen-focused therapy, BM-PCa patients also receive specific bone-targeted therapies to treat cancer-induced skeletal events that contribute to poor quality of life. Despite the extensive evidence of chemokine functions in the tumor-bone space, research is lacking on the impact of bone-targeted therapies on osteoid cell-derived chemokines. Since both bisphosphonates and denosumab primarily target osteoclasts, the greatest effect of bone-targeted therapies may be a reduction in osteoclast-derived chemokines. Yet, the contribution of osteoclasts to chemokine production in the BM-PCa microenvironment remains poorly studied. Osteoclasts are known to produce large amounts of CCL9 and slightly smaller amounts of other chemokines, including CCL22, CCL25, and CXCL13, but it is not reported whether BM-PCa upregulates any of these in osteoclasts (256). There is also limited research on the roles of some of these chemokines in BM-PCa. A single study reported that CCL22 induces BM-PCa cell migration, and one study observed that CCL25 promotes BM-PCa chemoresistance (257, 258). Further investigation is required to understand both the roles of osteoclast-derived chemokines in BM-PCa and the impact of bone-targeted therapies on those chemokines.

It is also possible that targeting of bone resorption may alter chemokine expression regulated by bone-sequestered growth factors. For example, TGFβ has been shown to upregulate osteoclast CXCL16 expression, which recruits osteoblasts (259). Reducing the amount of tumor-induced osteolysis would likely then result in fewer osteoblasts in the area, which would consequently reduce the levels of osteoblast-derived chemokines.

Additionally, a few studies have identified bisphosphonate treatment specifically to alter chemokine expression in other cancer types. In bronchoalveolar macrophages, the bisphosphonate zoledronate has been observed to promote upregulation of pro-inflammatory chemokines such as CCL2 and CCL5 in response to immune challenge (260). Yet, in osteosarcoma cells zoledronate downregulated CCL2 (219). However, in osteosarcoma and bone-metastatic breast and prostate cancer cells bisphosphonates downregulated CXCR4, which suppressed CXCL12-CXCR4 signaling-induced invasion though this phenomenon needs to be tested *in vivo* (261–263). These findings suggest bisphosphonates could be targeting BM-PCa by suppressing pro-tumoral signaling through CXCR4 in addition to blocking tumor-induced osteolysis. The impact of bisphosphonates on chemokine production appears to be cell type-specific and remains to be investigated in osteoid cells of the BM-PCa microenvironment.

It is likely that Denosumab, the monoclonal antibody of RANKL, also alters osteoid cell chemokine signaling in BM-PCa; however, the detailed mechanisms have yet to be discovered. RANKL downregulates expression of CCR2 and CCR5 on macrophages and CXCR6 and CX3CR1 on osteoclasts (256, 264, 265). If these receptors on tumor cells are similarly inhibited by RANKL, then inhibition of RANKL by denosumab would increase their expression and enhance pro-tumoral signaling by the osteoid cell-derived chemokines CCL2, CCL5, CXCL16, and CX3CL1. In this case, the addition of chemokine targeted treatments to denosumab treatment may be effective new combination therapies.

Another bone-targeted treatment, radium 223 dichloride, targets alpha radiation specifically to areas of active bone remodeling (266, 267) and will significantly impact osteoid cells, but changes in chemokine signaling have not been characterized. In the lungs, irradiated MSCs promote metastasis *via* upregulation of CCL5 (268). Other studies have observed CCL2 to be upregulated following radiation (269–271), but this has not been shown in osteoid cells to date. Therefore, while radium 223 likely suppresses tumor-induced bone formation by reducing osteoblast numbers and function, it may also upregulate pro-tumoral chemokines and osteolysis. Thus, the use of chemokine inhibitors and osteoclast-targeted therapies may block pro-tumoral responses to radium 223.

There is a major gap in understanding how bone targeted and standard of care hormonal therapy impacts chemokine signaling in BM-PCa. Additional research on the impact of bone-targeted therapies could further highlight potential combination treatment approaches for BM-PCa patients.

## Conclusion

In this review, we have discussed the importance of chemokines produced by osteoid cells that contribute significantly to PCa progression in the bone microenvironment. However, chemokine signaling in the tumor microenvironments is complex, and production of these chemokines by osteoid cells can also induce other cell types, including the cancer cells and recruited immune cells, to produce additional chemokines that can drive PCa progression. Clearly the research discussed in this review only covers a small part of the true impact of osteoid cell-derived chemokines on BM-PCa, as the ripple effect of many of these chemokines on other cell types and signaling pathways in the tumor microenvironment has yet to be elucidated.

Another area requiring further investigation is the dependence of BM-PCa on osteoid cell-derived chemokines since many of these chemokines can also be produced by the BM-PCa cells themselves (154, 254, 272–275). While it is likely the tumor cells simply take advantage of bone cell-produced chemokines to reduce the need to produce their own, it is also possible osteoid cells may primarily produce different isoforms of some of these chemokines which could differ in potency. For example, studies have shown that two different isoforms of CXCL8 are produced by different cell types and differ in potency (176, 276). The endothelial cell-derived isoform of CXCL8 is reported to have reduced potency in recruiting neutrophils compared to the isoform produced by monocytes and other cells (276). In breast cancer, two human cell lines were shown to each produce a different isoform of CXCL8 with a difference in potency between the two (176). Therefore, it is possible there may a difference in potency between PCa-derived and osteoid cell-derived isoforms of these chemokines. It is also possible that osteoid cells may produce more of these chemokines than the PCa tumor cells. In breast cancer, tumor cells were found to produce only picogram quantities of CCL2, whereas osteoblasts produced nanogram quantities of CCL2 (177). Additionally, it has been shown in osteosarcoma, a bone cancer arising from malignant transformation of osteoblasts, that tumor cell-derived CXCL8 can upregulate MSC expression of CXCL8, which then increases osteosarcoma cell expression of CXCL8, creating a positive feedback loop driving tumor progression (180). However, this has not been shown in PCa to date. Nonetheless, osteoid cell-derived chemokines play a key role in the progression of BM-PCa.

In summary, chemokines derived from osteoid cells in the bone microenvironment play key roles in driving PCa metastasis to bone and the progression of BM-PCa and represent promising new therapeutic targets and potential biomarkers.

## Author contributions

All authors listed have made a substantial, direct, and intellectual contribution to the work and approved it for publication.
